# HSPB5 Inhibition by NCI-41356 Reduces Experimental Lung Fibrosis by Blocking TGF-β1 Signaling

**DOI:** 10.3390/ph16020177

**Published:** 2023-01-24

**Authors:** Julie Tanguy, Pierre-Marie Boutanquoi, Olivier Burgy, Lucile Dondaine, Guillaume Beltramo, Burhan Uyanik, Carmen Garrido, Philippe Bonniaud, Pierre-Simon Bellaye, Françoise Goirand

**Affiliations:** 1 INSERM U1231, Faculty of Medicine and Pharmacy, University of Bourgogne-Franche Comté, 21000 Dijon, France; 2 UFR des Sciences de Santé, University of Bourgogne-Franche-Comté, 21000 Dijon, France; 3 Reference Center for Rare Pulmonary Diseases, University Hospital, Bourgogne-Franche Comté, 21000 Dijon, France; 4 Réseau OrphaLung, Filière RespiFIl, Department of Pulmonary Medicine and Intensive Care Unit, University Hospital, Bourgogne-Franche Comté, 21000 Dijon, France; 5 Cancer Center George François Leclerc, 21000 Dijon, France

**Keywords:** TGF-β, HSPB5, pulmonary fibrosis (PF), anti-fibrotic therapy

## Abstract

Idiopathic pulmonary fibrosis is a chronic, progressive and lethal disease of unknown etiology that ranks among the most frequent interstitial lung diseases. Idiopathic pulmonary fibrosis is characterized by dysregulated healing mechanisms that lead to the accumulation of large amounts of collagen in the lung tissue that disrupts the alveolar architecture. The two currently available treatments, nintedanib and pirfenidone, are only able to slow down the disease without being curative. We demonstrated in the past that HSPB5, a low molecular weight heat shock protein, was involved in the development of fibrosis and therefore was a potential therapeutic target. Here, we have explored whether NCI-41356, a chemical inhibitor of HSPB5, can limit the development of pulmonary fibrosis. In vivo, we used a mouse model in which fibrosis was induced by intratracheal injection of bleomycin. Mice were treated with NaCl or NCI-41356 (six times intravenously or three times intratracheally). Fibrosis was evaluated by collagen quantification, immunofluorescence and TGF-β gene expression. In vitro, we studied the specific role of NCI-41356 on the chaperone function of HSPB5 and the inhibitory properties of NCI-41356 on HSPB5 interaction with its partner SMAD4 during fibrosis. TGF-β1 signaling was evaluated by immunofluorescence and Western Blot in epithelial cells treated with TGF-β1 with or without NCI-41356. In vivo, NCI-41356 reduced the accumulation of collagen, the expression of TGF-β1 and pro-fibrotic markers (PAI-1, α-SMA). In vitro, NCI-41356 decreased the interaction between HSPB5 and SMAD4 and thus modulated the SMAD4 canonical nuclear translocation involved in TGF-β1 signaling, which may explain NCI-41356 anti-fibrotic properties. In this study, we determined that inhibition of HSPB5 by NCI-41356 could limit pulmonary fibrosis in mice by limiting the synthesis of collagen and pro-fibrotic markers. At the molecular level, this outcome may be explained by the effect of NCI-41356 inhibiting HSPB5/SMAD4 interaction, thus modulating SMAD4 and TGF-β1 signaling. Further investigations are needed to determine whether these results can be transposed to humans.

## 1. Introduction

Idiopathic pulmonary fibrosis (IPF) is a chronic, progressive and fatal disease of unknown etiology with a median of survival of only 4–5 years that mainly affects individuals over 60 years old [1,2]. IPF is characterized by the excessive and aberrant deposition of extracellular matrix (ECM) in the lung parenchyma, resulting in impaired respiratory functions and progressive morbidity by pulmonary insufficiency. The latest estimates show an increase in the incidence of IPF worldwide with a global prevalence of 0.33–2.51 per 10,000 individuals in Europe [3]. Currently, there is no curative treatment for IPF and the available pharmacological treatments, pirfenidone and nintedanib, slow down the progression of the disease but do not stop it. While the mechanisms involved in IPF are not fully understood, the emergence and persistence of myofibroblasts responsible for the accumulation of ECM inside the lung are key components explaining the development and progression of IPF. The origin of the activation of these myofibroblasts is consequent to a lesion of alveolar epithelial cells promoted by environmental exposure, genetic modulation and/or aging [4]. Transforming growth factor (TGF)-β1 is a key pro-fibrotic factor involved in pulmonary fibrosis through the activation of the SMAD pathway [5]. Heat shock proteins (HSPs) are chaperone proteins involved in several pathways which ensure cellular homeostasis. HSPs have been shown to be involved in several pathological contexts including lung fibrosis [6]. Our team has shown that HSPB5 (also called αB-crystallin), a member of the small heat-shock proteins (sHSP) family, was up-regulated in experimental models of lung fibrosis and in IPF patients [7,8]. In IPF patients, we have demonstrated that HSPB5 is overexpressed in the active sites of fibrosis, more precisely in areas enriched with ECM-producing cells, myofibroblasts, but also in hyperplastic alveolar epithelial cells surrounding fibroblastic foci [7]. Moreover, HSPB5 interacts with SMAD4 and stabilizes the complex SMAD2/3/4 in the nucleus, thus promoting TGF-β1 signaling and subsequent lung fibrosis [7,8]. In addition, in HSPB5 knockout mice where the canonical Smad pathway is inhibited, pulmonary fibrosis was reduced [7,8].

In this context, HSPB5 seems to be a relevant target for the development of effective anti-fibrotic therapies. Last decade, Chen et al. discovered a small molecular inhibitor of HSPB5, NCI-41356 ((2S,3R)-3-Methylglutamic Acid Hydrochloride Salt), which inhibits the interaction of HSPB5 with its chaperoned partners [9]. In the context of breast cancer, the authors showed that NCI-41356 inhibited tumor cell proliferation and invasiveness and reduced tumor growth in a preclinical cancer model [9]. Fibrosis and cancer share several properties such as genetic alterations, uncontrolled cell proliferation, altered cell interaction and communication and tissue invasion [10]. To our knowledge, the pharmacological inhibition of HSPB5 to treat lung fibrosis has never been explored so far. 

The aim of this work is to demonstrate the interest of targeting HSP5 in progressive pulmonary fibrosis by examining in vivo and in vitro the HSPB5 inhibitor NCI-41356.

## 2. Results

### 2.1. Systemic Intravenous Administration of NCI-41356 Protects from Bleomycin (BLM) Induced Lung Fibrosis

**Figure 1:**
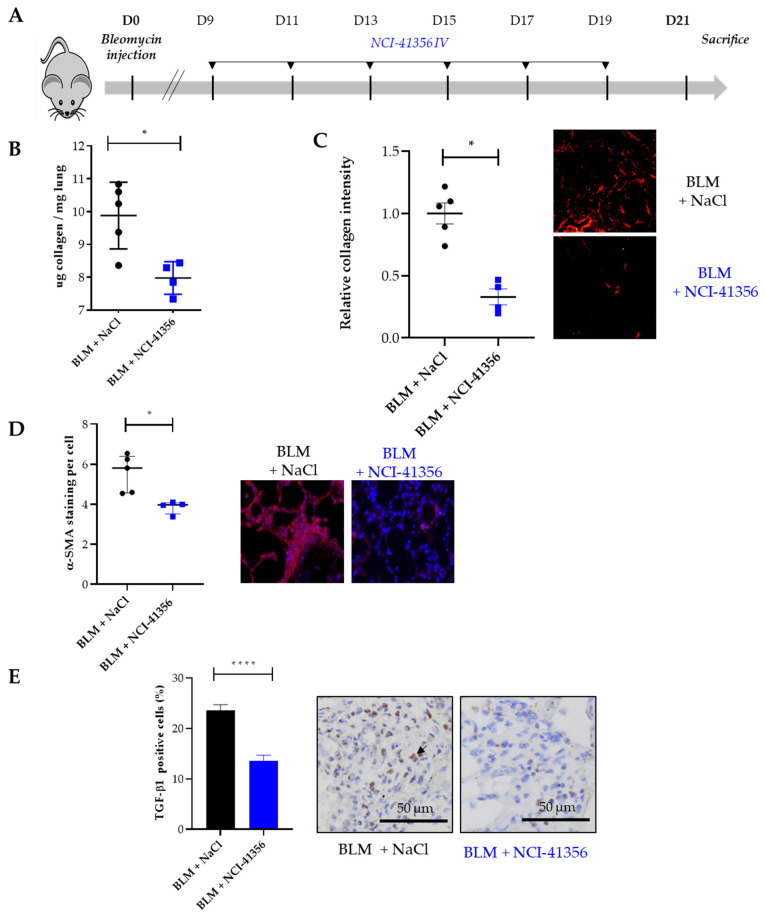
Intravenous administration of NCI-41356 reduces pulmonary fibrosis in bleomycin treated mice. (**A**). Mice experimental injection plan between day 0 (D0), the day of bleomycin (BLM) injection (1.5 mg/kg) and the day of sacrifice (D21). Mice were treated with NaCl 0.9% (*n* = 6) or NCI-41356 (*n* = 6) (15 mg/kg) 3 times a week between day 9 and 21; (**B**). Collagen quantification by Sircol assay (expressed as µg of collagen per mg of lung) of bleomycin treated mice lungs treated or not with NCI-41356 (* *p* = 0.0317); (**C**). Collagen semi-quantification by Sirius Red staining performed on lung sections of mice treated by bleomycin with or without NCI-41356 (* *p* = 0.0159, control = 1); (**D**). Quantification of α-SMA staining by immunofluorescence performed on lung sections of mice treated by bleomycin with or without NCI-41356 (* *p* = 0.0159); (**E**). TGF-β1 mRNA expression (brown color, black arrow) on lung tissues of mice treated by bleomycin with or without NCI-41356 (**** *p* < 0.0001)(×20). Data are expressed as means ± SEM and compared using nonparametric Mann–Whitney test (Figure 1B–D) or Welch’s test (Figure 1E).

Lung fibrosis was induced by intra-tracheal (i.t.) administration of BLM at day 0 (D0). In our model, fibrosis began to appear between day 7 (D7) and day 9 (D9). HSPB5 inhibitor NCI-41356 was administered during fibrosis progression from D9 to D21 by intravenous (i.v.) injection (Figure 1A). Interestingly, NCI-41356 significantly decreased the accumulation of collagen in the lungs at D21 (Figure 1B, * *p* = 0.0317) compared with mice receiving vehicle (NaCl). Collagen quantification was confirmed by histological measurements that also showed a decrease in collagen lung content at D21 upon NCI-41356 treatment (Figure 1C, * *p* = 0.0159). Consistently with these results, tissue remodeling of BLM-treated animals was significantly greater than that of NCI-41356 treated animals (Supplemental Figure S1, * *p* = 0.0159). Moreover, NCI-41356 was able to effectively reduce the expression of α-SMA, one important marker of myofibroblast differentiation (Figure 1D, * *p* = 0.0159). Furthermore, we demonstrated by RNAscope that NCI-41356 significantly reduced TGF-β1 mRNA expression in the lung tissue of mice treated with BLM (Figure 1E, **** *p* < 0.0001). 

### 2.2. Local Intra-Tracheal Administration of NCI-41356 Protects from BLM-Induced Lung Fibrosis

**Figure 2:**
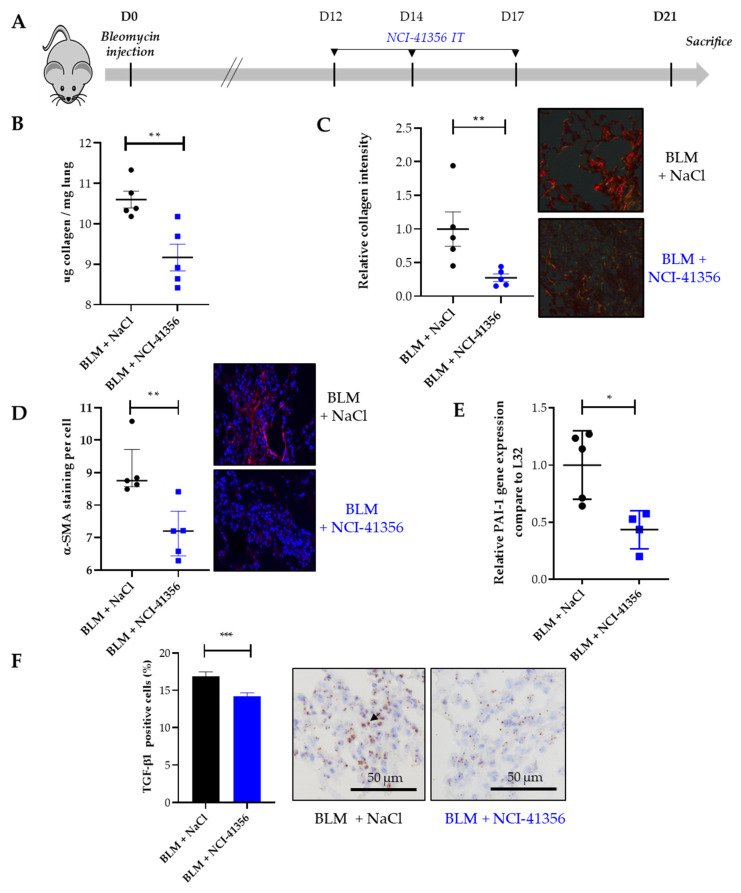
Intratracheal administration of NCI-41356 reduces pulmonary fibrosis in bleomycin-treated mice. (**A**). Mouse experimental injection plan between day 0 (D0), the day of bleomycin (BLM) injection (1.5 mg/kg) and the day of sacrifice (D21). Mice were treated with NaCl 0.9% (*n* = 6) or NCI-41356 (*n* = 6) (15 mg/kg) at D12, D14 and D17; (**B**). Collagen quantification by Sircol assay (expressed as µg of collagen per mg of lung) of mice lungs treated with bleomycin with or without NCI-41356 (** *p* = 0.0079); (**C**). Collagen semi-quantification by Sirius Red staining performed on lung sections of mice treated by bleomycin with or without NCI-41356 (** *p* = 0.0079, control = 1) and representative staining; (**D**). Quantification of α-SMA staining by immunofluorescence performed on lung tissues of mice treated by bleomycin with or without NCI-41356 (** *p* = 0.0079); (**E**). Relative PAI-1 mRNA expression in the lung of mice treated by bleomycin with or without NCI-41356 (* *p* = 0.0159); (**F**). TGF-β1 mRNA expression (brown color, black arrow) on lung tissues from mice treated by bleomycin with or without NCI-41356 (*** *p* = 0.0002) (×20). Data are expressed as means ± SEM and compared using nonparametric Mann–Whitney test (Figure 2B–E) or Welch’s test (Figure 2F).

Using the BLM mouse model of lung fibrosis described above, we decided to try another route of injection of our compound, more relevant to locally treat already established fibrosis. In this experiment, mice received three intra-tracheal (i.t.) treatments with NCI-41356 at D12, D14 and D17 (Figure 2A). Intratracheal NCI-41356 was able to decrease collagen accumulation and limit tissue remodeling in lungs upon BLM (Figure 2B,C, ** *p* = 0.0079 and ** *p* = 0.0079; Supplemental Figure S1, * *p* = 0.0159) compared to vehicle. In addition, intratracheal NCI-41356 reduced α-SMA (Figure 2D, ** *p* = 0.0079) and PAI-1 expression (Figure 2E, * *p* = 0.0159). Using RNAscope, we demonstrated that intratracheal NCI-41356 also induced a decrease in TGF-β1 mRNA expression in lungs upon BLM (Figure 2F, *** *p* = 0.0002) compared to vehicle. 

### 2.3. NCI-41356 Inhibits HSPB5 Activity and Prevents HSPB5/SMAD4 Interaction

**Figure 3:**
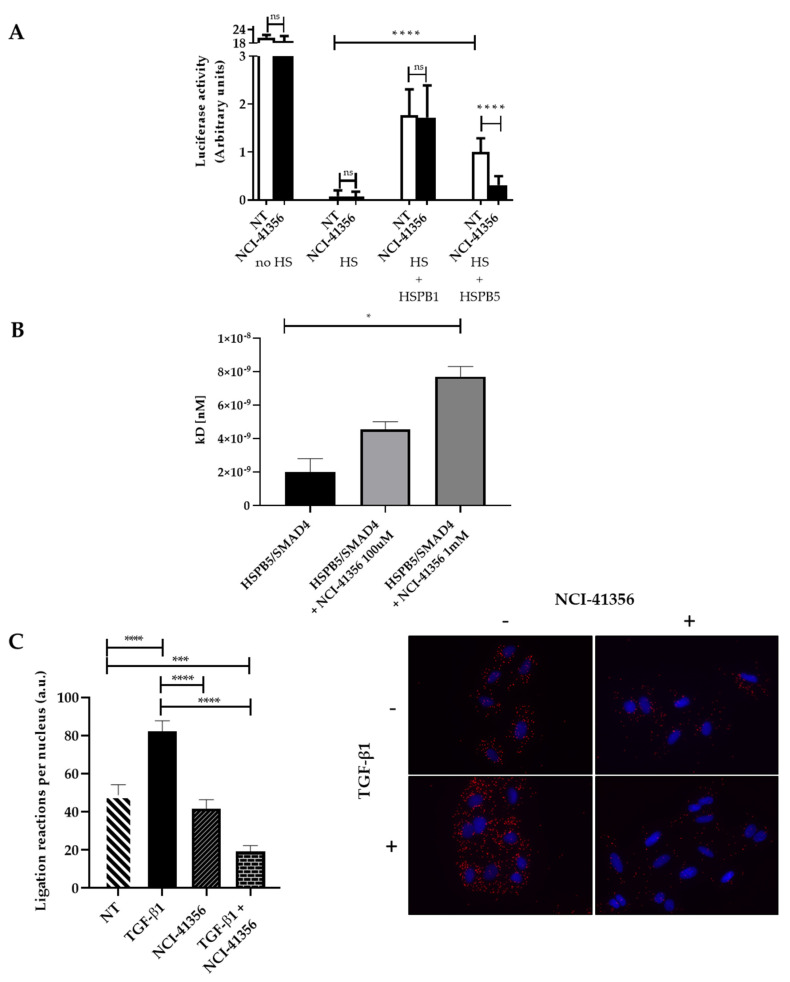
NCI-41356 decreases SMAD4/HSPB5 interaction. (**A**). Anti-aggregation assay on luciferase exposed to heat shock (HS) in presence or absence of recombinant chaperone HSPB1/HSPB5 (1.5 μM) and NCI-41356 (100 µM) (*n* = 3) (**** *p* < 0.0001); (**B**). Interaction measurement by thermophoresis between HSPB5 and SMAD4 proteins in presence or not of NCI-41356, tested at 100 µM and 1 mM (* *p* = 0.0107); K_D_ (dissociation constant); (**C**). Proximity ligation assay between SMAD4/HSPB5 on A549 cells treated by either NCI-41356 (100 µM) or TGF-β1 (10 ng/mL) or both compounds (quantification one the left and representative image on the right) (×40); One red spot matches with one interaction between these two proteins (*n* = 3) (*** *p* = 0.0003; **** *p* < 0.0001). Data are expressed as means ± SEM and compared using nonparametric Mann–Whitney (anti-aggregation assay) and Kruskal–Wallis tests (thermophoresis assay) or one-way ANOVA (PLA assay).

**Figure 4:**
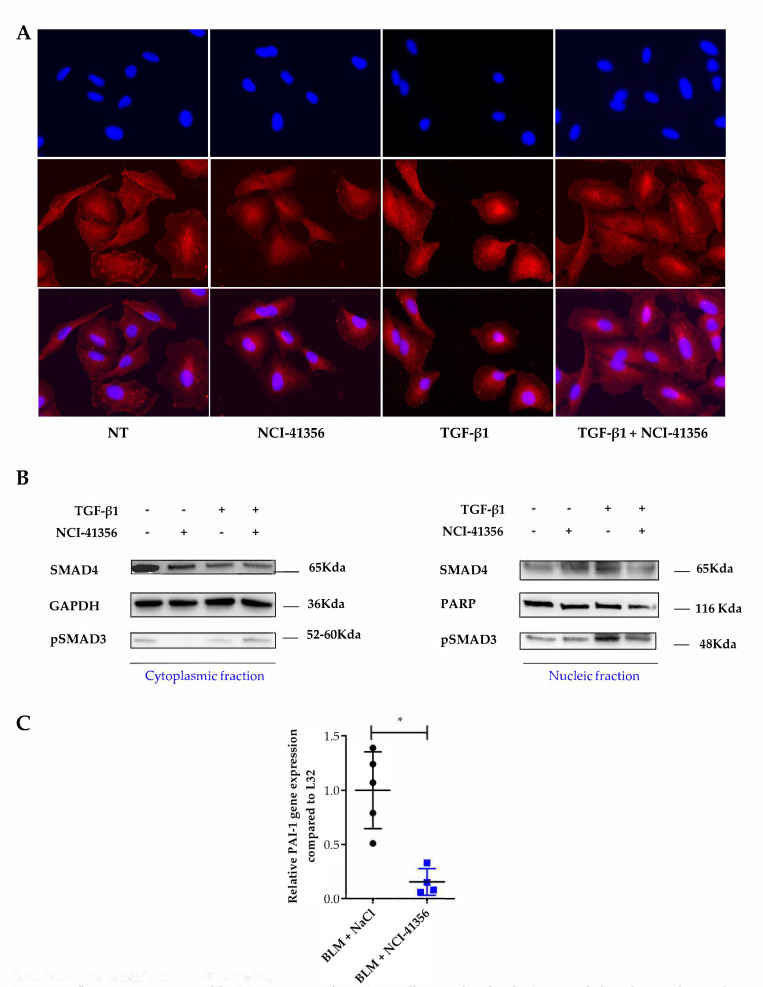
NCI-41356 modulates TGF-β1 signaling. (**A**). Immunofluorescence staining of SMAD4 protein (red) on A549 cells treated with TGF-β1 (10 ng/mL) with or without NCI-41356 (100 µM) (*n* = 3) (×40). Nuclear staining: 4′,6-diamidino-2-phenylindole (blue); (**B**). Protein expression os Smad4, pSMAD3, GAPDH and PARP studied by Western blot of nuclear or cytoplasmic fractions of A549 cells treated with TGF-β1 (10 ng/mL) with or without NCI-41356 (100 µM). One representative experiment out of three is shown; (**C**). Relative PAI-1 mRNA expression compared to control L32 in the lung of mice treated by bleomycin with or without NCI-41356 by intravenous route (* *p* = 0.0159). Data are expressed as means ± SEM and compared using nonparametric Mann–Whitney test.

HSPs are chaperones that prevent protein misfolding and aggregation. We first studied whether NCI-41356 was able to inhibit the chaperone activity of HSPB5. The capacity of a heat shock (HS) to denature luciferase in the presence or absence of HSPB5 or HSPB1 (as negative control) and of NCI-41356 was determined. As expected, in absence of NCI-41356, both HSPB1 and HSPB5 were able to partially restore luciferase activity upon HS (Figure 3A, **** *p* < 0.0001). However, in the presence of NCI-41356, the protective activity of HSPB5 on luciferase was significantly decreased (Figure 3A, **** *p* < 0.0001). On the contrary, NCI-41356 had no effect on the activity of its closely related chaperone HSPB1, demonstrating the specificity of the inhibitor for HSPB5. In vitro, we determined by a thermophoresis interaction assay that the affinity between HSPB5 and its partner SMAD4 was altered in the presence of NCI-41356 in a dose-dependent manner (Figure 3B, * *p* = 0.0107). HSPB5 has been shown to interact with SMAD4 to favor its nuclear localization in profibrotic conditions [7]. Using a proximity ligand assay (PLA) experiment, we demonstrated an increase in the proximity between SMAD4 and HSPB5 upon TGF-β1 which was inhibited by NCI-41356 (Figure 3C, **** *p* < 0.0001). Furthermore, in A549 epithelial cells, we demonstrated that NCI-41356 was able to inhibit the translocation of the complex SMAD3/4 into the nucleus induced by TGF-β1 (Figure 4A,B and Supplemental Figure S2). Consistently, in our murine lung fibrosis model, intravenous NCI-41356 decreased the expression of PAI-1, an activation marker of TGF-β1 signaling (Figure 4C, * *p* = 0.0159).

**Figure 5:**
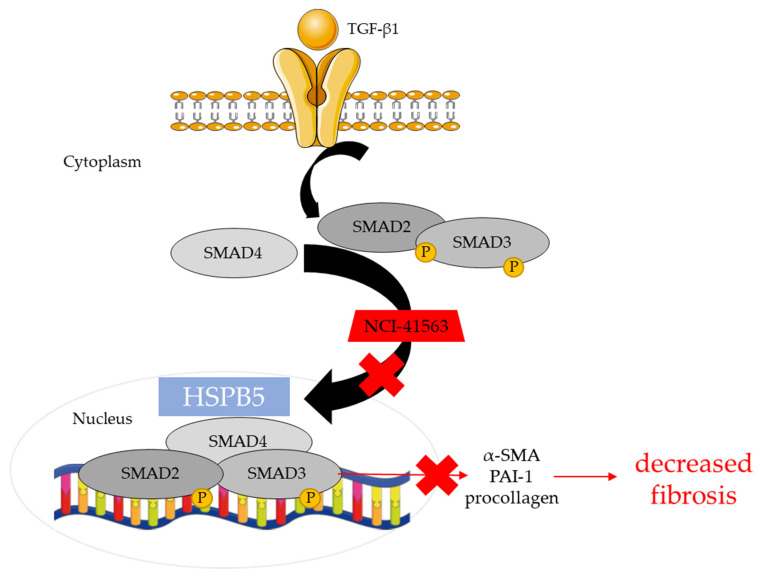
Proposed mechanical pathway involved in inhibitory effects of NCI-41356. NCI-41356 inhibits the interaction between HSPB5 and SMAD4, thus hampering the translocation of the SMAD4/pSMAD2/pSMAD3 complex into the nucleus. As a consequence, NCI-41356 limits the synthesis of collagen, pro-fibrotic markers and subsequent fibrosis.

Altogether, our results demonstrate that NCI-41356 is able to inhibit the progression of pulmonary fibrosis when it is injected locally (intratracheal) or systemically (intravenous) by interfering with TGF-β1 signaling (Figure 5).

## 3. Discussion

The lack of curative therapy for IPF remains a major clinical issue and it is the driving force behind our effort to find novel therapeutic strategies with compounds affecting different pro-fibrotic pathways. HSPs have been shown to play a crucial role in disease development and progression in cancer [11], but also in fibrotic disorders [6,12]. In the context of fibrosis, most HSPs have been shown to be involved in the modulation of TGF-β1 signaling, one of the main pro-fibrotic factors driving IPF progression [6]. This suggests that the combination of HSP inhibitors with nintedanib or pirfenidone, which affect other pro-fibrotic pathways, could be beneficial. The recent interest in HSPs has led to the development of numerous inhibitors that are now being tested in clinical trials, mainly in patients with cancer [13]. While clinical trials have mainly focused on HSPC1 blockade in cancer, inhibitors of other HSPs have recently emerged [14]. Chen et al. used structure-based molecular docking of HSPB5 and identified a potent small molecular inhibitor, NCI-41356 [9]. NCI-41356 also inhibited tumor growth and vasculature development in breast cancer xenograft models [9]. Our study confirms the findings by Chen et al. by demonstrating that NCI-41356 is able to specifically inhibit HSPB5 chaperone activity. Most interestingly, NCI-41356 did not inhibit the chaperone activity of HSPB1, another small HSP close to HSPB5, further demonstrating the specificity of this inhibitor for HSPB5. Chen et al. focused their study on the interaction between HSPB5 and vascular endothelial growth factor (VEGF) in the context of cancer, and considering previous data from our team and others on the role of the SMAD-dependent TGF-β1 pathway in pulmonary fibrosis and on the role of HSPB5 in this pathway by chaperoning SMAD4 [7], we focused our study on the impact of NCI-41356 on HSPB5/SMAD4 interaction. We demonstrated that NCI-41356 was able to disrupt the nuclear interaction of HSPB5 and SMAD4 in a fibrotic context, thus limiting the translocation of SMAD4 and his partner pSMAD3 into the nucleus and leading to the inhibition of TGF-β1 signaling in vitro and lung fibrosis in vivo. Our results highlight a novel mechanism of action of this inhibitor on the SMAD-dependent TGF-β1 pathway, in addition to the already reported anti-cancer role of NCI-41346 on the VEGF pathway [9]. In addition, our results are in accordance with previous studies demonstrating that the lack of HSPB5 in knock-out mice disrupted the TGF-β1 pathway by interfering with SMAD4 nuclear localization [7,8,15]. Our study therefore strengthens the concept of the anti-fibrotic role of HSPB5 inhibition. Indeed, compared to previous studies that were performed on HSPB5 knock-out mice in which HSPB5 was deleted before the induction of lung fibrosis, our study demonstrates the anti-fibrotic role of HSPB5 inhibition with NCI-41356 administered at the later stages of bleomycin-induced fibrosis (D9-D12) when fibrosis is already initiated and progressive. This is particularly crucial as IPF patients are usually diagnosed at mild to advanced stages of the disease, at which fibrotic patches are already present in the lungs. 

In our study we identified that, in the context of lung fibrosis, the inhibition of HSPB5 by NCI-41356 interfered with the TGF-β1 pathway through SMAD3/4. TGF-β1 is a cytokine having pleiotropic features outside of its role in fibrosis. TGF-β1 signals are involved in a vast array of physiological and pathological processes, such as development, control of immunity and inflammation, cancer and fibrosis. Although there has been a lot of research into the development of direct TGF-β1 inhibition strategies, results from clinical trials, mainly in cancer, have been discouraging and associated with severe side effects [16]. Due to TGF-β1’s widespread impacts, the benefit/side effects balance of long-term inhibition is problematic [17]. The specificity of NCI-41356 to target the role of HSPB5 on SMAD3/4 signals may limit side effects that would result from direct inhibition of TGF-β1, while still inhibiting the SMAD-dependent pro-fibrotic pathways.

We cannot completely rule out the possibility that other partners of HSPB5, which may be affected by NCI-41356, might also directly or indirectly contribute to the overall antifibrotic effect of this HSPB5 inhibitor. VEGF is one of HSPB5′s partners identified by Chen et al. that, although having a controversial effect in fibrosis development [18], may be worth exploring.

The efficacy of a treatment is highly dependent on the route of administration and on the ability to achieve the optimum concentration of the drug in the targeted organ, producing a therapeutic benefit without inducing severe side effects. Due to their crucial role in cellular homeostasis, systemic inhibition of HSPs often comes with undesirable side effects and unacceptable toxicity [19]. Similarly, with TGF-β1 being a multifunctional cytokine of key importance in the maintenance of tissue homeostasis, systemic targeting of TGF-β1 signaling has been associated with severe off-target side effects [20]. Even though systemic administration is the most common route of drug delivery, in the context of IPF, local pulmonary administration of therapeutic compounds may circumvent the occurrence of adverse side effects, while optimizing the drug concentration delivered directly to the site of active fibrosis. In this way, we can drive the drug towards local immune cells like macrophages as well as the alveolar epithelial cells (AECs), which play a prominent role in pulmonary fibrosis [21,22]. Pulmonary drug delivery for IPF has gained interest in the last decade. Inhaled therapy with TD139 (a novel and potent small-molecule inhibitor of Galectin (Gal)-3 a profibrotic β-galactoside-binding lectin that plays a key role in the pathogenesis of IPF and IPF exacerbations) has been recently tested in IPF patients with encouraging results [23]. Moreover, inhaled formulations of both pirfenidone and nintedanib have been successfully tested [24,25]. For both drugs, local administration in the lungs was associated with higher lung and lower plasma concentrations with a conserved anti-fibrotic efficacy [24,25]. Here, we show strong anti-fibrotic effects of locally administered (i.t.) NCI-41356 in mice, even when fibrosis is already well established (i.e., starting at D12 with three administrations up to D21). Interestingly, this effect is comparable to that observed after systemic administration of the HSPB5 inhibitor at earlier stages of fibrosis (i.v. starting at D9 with six injections up to D21), despite the fact that local administration involved fewer injections (three i.t. versus six i.v. NCI-41356 administrations, respectively). 

These results suggest a potential interest of this drug as an inhaled therapy for patients with IPF, for whom therapies often arrive once fibrosis is already well established.

Nevertheless, drug delivery to the lungs does not come without issues. Inhalable formulations are a challenge and drug absorption, particle size, inertia, gravitational sedimentation and Brownian motion are issues that need to be taken into account [26]. Further studies are warranted in order to establish the feasibility of using NCI-41356 as an inhaled aerosol to treat lung fibrosis.

## 4. Materials and Methods

### 4.1. Animal Procedures

All animals were maintained in specific pathogen-free facilities in accordance with FELASA and Animal Experimental Ethics Committee guidelines (protocol code number 20745, University of Burgundy, France). This study complied with all relevant ethical regulations for animal testing and research and received ethical approval from the Animal Experimental Ethics Committee (University of Burgundy, France). Experiments were performed on 8-week-old female C57BL/6 mice weighing 20 to 25 g (Charles River, Saint Germain-sur-l’Arbresle, France). Intratracheal instillation of bleomycin (BLM) at 1.5 mg/kg (Santa Cruz Biotechnology, Dallas, TX, USA) or NaCl (control) was performed as previously described [27]. Mice were regularly weighed and supplemented with Gel Diet (SAFE gel diet, France). (2S,3R)-3-Methylglutamic acid hydrochloride salt (NCI-41356) (sc-206572, Santa Cruz) or NaCl (vehicle) were instilled at 15 mg/kg by intratracheal injection (at day 12, 14 and 17) or by intravenous injection every other day from day 9 to 19 post-BLM. Mice were euthanized by abdominal aortic bleeding on day 21 post-BLM. Lungs were collected for histological, transcriptomics and collagen quantification analyses. 

### 4.2. Collagen Quantification

For histomorphometric assay, the amount of collagen in paraffin-embedded tissue sections was quantified on lung slices from BLM- or NaCl-receiving mice treated or not with NCI-41356 after staining with Picro sirius Red as previously described [28].

For colorimetric assay, the Sircol assay was performed on lung left lobe extracts using the Sircol kit (Biocolor Ltd., Carrickfergus, UK) and following the manufacturer’s recommendations. Results were expressed per mg of lungs tissue.

### 4.3. Ashcroft Scoring

Pulmonary fibrosis was graded using a modified Ashcroft score [29]. Each lung section was scanned and graded from 0 (normal lung) to 8 (completely fibrotic lung) by three different blinded experimenters. The mean value of the grades obtained for all of the fields was used as the fibrotic score.

### 4.4. Duolink Proximity-Ligation Assay (PLA)

PLA assay was performed with Duolink In Situ Orange Starter Kit Mouse/Rabbit (DUO92102-1KT, Sigma Aldrich) following the manufacturer’s recommendations. The primary antibodies used were rabbit anti-SMAD4 (ab40759, 1:100, Abcam) and mouse anti-HSPB5 (ADI-SPA-222, 1:100, Enzolife). Microscopy images were taken on an Axio Imager 2 (Carl Zeiss Microscopy GmbH, Jena, Germany). Images were acquired using an AxioCam MRm monochrome CCD camera (Carl Zeiss GmbH) and analyzed using ImageJ software.

### 4.5. Immunofluorescence

Cells seeded on coverslips in 12-well plates were treated with TGF-β1 (10 ng/mL) and/or NCI-41356 (100uM) for 24 h. Cells were then fixed in 4% formaldehyde for 15 min at room temperature (RT), washed and permeabilized with 100% methanol for 20 min on ice. Then, cells were blocked for 1 h with TBS-T 3% BSA and incubated with primary antibody at 4 °C overnight (anti-SMAD4, ab40759, 1:100, Abcam, Paris, France). After incubation, cells were washed and stained with secondary fluorescent antibodies (CF Dye 568; Biotium, Fremont, CA, USA). Finally, coverslips were rinsed three times with 1X PBS, stained with DAPI (Sigma-Aldrich, Saint-Quentin-Fallavier, France) and mounted on slides with Fluoromount-G mounting medium (Southern Biotech, Birmingham, AL, USA). Images were acquired as described above and analyzed by Zen 2.1 Lite and ImageJ software 1.5.

Lung tissue previously included in paraffin were deparaffinized, blocked for 1 h with TBS-T 3% BSA and incubated with primary antibody at 4 °C overnight (anti-α-SMA, ab5694, 1:100, Abcam). After incubation, lung sections were washed and stained with secondary fluorescent antibodies (CF Dye 568; Biotium, Fremont, CA, USA). Finally, sections were rinsed three times with 1X PBS, stained with DAPI (Sigma-Aldrich, Saint-Quentin-Fallavier, France) and mounted on slides with Fluoromount-G mounting medium (Southern Biotech, Birmingham, AL, USA). Images were acquired as described above and analyzed by Zen 2.1 Lite and ImageJ software 1.5.

### 4.6. RNAscope Assay

TGF-β1 mRNA expression was measured using RNAscope assay (RNAscope™ 2.5 HD Assay – BROWN, ACDbio, Abingdon, United Kingdon). The assay was performed according to the manufacturer’s recommendations. Briefly, lung sections previously included in paraffin were deparaffinized and endogenous peroxidase activity was blocked before slides were boiled in the 1X Target Retrieval solution for 15 min. Slides were then treated with RNAscope^®^ Protease Plus solution and incubated in a hybridization chamber (HybEZ™ Oven, Advanced Cell Diagnostics, Newark, CA, USA) for 30 min at 40 °C. Next, slides were incubated with Mm-TGF-β1 probes (407751 ACD bio) or positive or negative control probes (m-PPIB or DapB) at 40 °C for 2 h in a HybEZ™ Oven. Later, amplification reagents (Amp-1–6) were sequentially added to the samples and finally sections were incubated with Fast RED-B and Fast RED-A solutions before counterstaining in hematoxylin solution. Then, they were covered with cover slips using EcoMount medium (biocare medical EM897L).

### 4.7. RNAscope Quantification

Microscopy images were taken on an Axio scope.A1 (Carl Zeiss GmbH, Oberkochen, Germany). Images were acquired using Gryphax Jenoptik software and analyzed as previously described using Qupath software (version 0.2.3) [30]. For each slide, 20 randomly taken pictures at x20 were analyzed. Stain vectors were defined by selecting a region of interest that was representative of the stain. The positive cell detection feature was utilized to identify the positive cells and each automated detection was manually verified. To facilitate visualization, cells were colored on the basis of whether or not they were positive for the marker. The number of positive and negative cells in each specimen for each section was then quantified and results are expressed as the percentage of TGFβ-1-positive cells for each specimen.

### 4.8. Protein Interaction Studies

Protein HSPB5 (Enzolife, ADI-SPP-228-200) was labeled using the Protein Labeling Kit RED-Tris-NTA (NanoTemper Technologies). The labeling reaction was performed according to the manufacturer’s instructions in the supplied labeling buffer, applying a concentration of 1.6 μM protein (molar dye: protein ratio ≈ 1:2) at room temperature for 30 min in the dark. Unreacted dye was removed by centrifugation. The degree of labeling was determined using UV/VIS spectrophotometry at 650 and 280 nm. A degree of labeling of 0.8 was typically achieved. The labeled protein HSPB5 was adjusted to 400 nM with PBS buffer supplemented with 0.05% Tween 20. The ligand SMAD4 (ABIN1065691) was dissolved in PBS buffer supplemented with 0.05% Tween 20 and a series of 16 1:1 dilutions was prepared using the same buffer. For the measurement, each ligand dilution was mixed with one volume of labeled protein HSPB5, which led to a final concentration of protein HSPB5 of 104 nM and final ligand concentrations ranging from 19 pM to 15.6 μM. After 10 min of incubation followed by centrifugation at 10,000× *g* for 10 min, the samples were loaded into Monolith NT.115 Capillaries (NanoTemper Technologies, München, Germany). MST was measured using a Monolith NT.115 instrument (NanoTemper Technologies, München, Germany) at an ambient temperature of 25 °C. Instrument parameters were adjusted to 100% LED power and medium MST power. Data of three independently pipetted measurements were analyzed (MO.Affinity Analysis software version 2.3, NanoTemper Technologies) using the signal from an MST and a time of 1s. K_D_ (dissociation constant) = $\frac{1}{KA\;\left(affinity\;constant\right)}$

### 4.9. Cell Fractionation and Western Blot Analysis

Cytoplasmic and nuclear extracts were obtained using the NE-PER Nuclear and Cytoplasmic Extraction reagent kit (Pierce–Thermo Scientific, Rockford, IL, USA). Samples were prepared in a classical loading buffer and loaded on SDS-PAGE gel before being transferred onto a PVDF membrane (GE Healthcare Europe GmbH) in an ethanol-supplemented glycin buffer. Membranes were saturated with TBS-Tween 0.1% Milk 5% solution for 45 min before overnight incubation at 4 °C with primary antibodies: rabbit SMAD4 (40759, 1:1000, Abcam), rabbit GAPDH (5174, 1:1000 Cell Signaling), rabbit PARP (9542,1:1000 Cell Signaling), rabbit Phospho-Smad3 (Ser423/425) (D27F4) (#8828, 1:1000, cell signaling) and rabbit HSPB5 (ADI-SPA-223, 1:1000 enzolife). Secondary antibodies used for revelation were conjugated to HRP (Jackson ImmunoResearch, Cambridge, UK). Data acquisition was performed using a chemilumiscent reagent (Western Blotting Luminol Reagent. Santa Cruz Biotechnology) on Chemi-Doc XRS Imaging System (Bio-Rad). 

### 4.10. Quantitative PCR Analysis

Total RNA from lung tissue was extracted with Trizol (Invitrogen, Waltham, MA, USA). In all, 300 ng of total RNA was transcribed into cDNA by M-MLV reverse transcriptase with random primers in the presence of RNaseOUT RNAse inhibitor (Invitrogen). cDNAs were quantified by real-time PCR with a SYBR Green Real-time PCR kit (Applied Biosystems) on a Viaa7 detection system (Applied Biosystems, France). Relative mRNA levels were determined with the ΔCt method. Oligonucleotides used for qRT-PCR are as follows: PAI-1, 5′-GGCCGTGGAACAAGAATGAGA-3′ and 5′-GCTTGAAGAAGTGGGGCATGA-3′; L32, 5′-GAAACTGGCGGAAACCCA-3′ and 5′-GGATCTGGCCCTTGAACCTT-3.

### 4.11. Anti-Aggregation Activity of HSPB5

HSPB5 anti-aggregation function was evaluated as previously described [31]. Human HSPB5 (ADI-SPP-228, Enzolife, Villeurbanne, France) and human HSPB1 (ADI-SPP-715, Enzolife, Villeurbanne, France) (1.5 μM) were incubated with firefly luciferase (Sigma-Aldrich, L9506) in reaction buffer (HEPES-KOH 25 mM pH 7.6, MgCl_2_ 5 mM, DTT 2 mM, ATP 2 mM) in the presence or absence of NCI-41356 (100 μM) and heated in a water bath for 30 min at 42 °C. The heated reaction was diluted 10-fold into buffer containing 60% rabbit reticulocyte lysate (TnT^®^T7 QuickMaster Mix, Promega) and incubated for 2 h at RT. Anti-aggregation activity was assessed through a luciferase assay on Envision system (Perkin Elmer, Villebon-sur-Yvette, France).

### 4.12. Statistical Analysis

All results are shown as means ± SEM and data sets were compared using nonparametric Mann–Whitney test or parametric Welch’s test to compare two groups, or non-parametric Kruskal–Wallis test or one-way ANOVA for multiple comparison. Statistical analysis was performed with GraphPad Prism 8.3. All *p* values were two-tailed. A *p*-value of <0.05 was considered statistically significant for all experiments.

## 5. Conclusions

In our study, we demonstrate the anti-fibrotic potential of NCI-41356 as a new chemical inhibitor of HSPB5/SMAD4 interaction in a bleomycin-induced pulmonary fibrosis model. Our results show that NCI-41356 can limit collagen accumulation in the lungs and decrease the expression of several pro-fibrotic markers through the modulation of TGF-β1 signaling. Our findings suggest that NCI-41356 could constitute an effective therapeutic strategy in IPF.

## Data Availability

Data is contained within the article and supplementary material.

## Supplementary Materials

The following supporting information can be downloaded at: https://www.mdpi.com/article/10.3390/ph16020177/s1, Figure S1: Ashcroft analysis of pulmonary fibrosis induced in bleomycin-treated mice treated or not with NCI-41356 by intra-tracheal (IT) or intravenous (IV) routes of administration. Figure S2: Immunofluorescence nucleic SMAD4 quantification evaluated on cells stimulated by TGF-β1 and treated by NCI-41356.
