# Supplementary material for: HSPB5 Inhibition by NCI-41356 Reduces Experimental Lung Fibrosis by Blocking TGF-β1 Signaling
Source: Pharmaceuticals (Basel). 2023 Jan 24;16(2):177. doi: 10.3390/ph16020177 (PMC9960643; doi:10.3390/ph16020177)
Supplement: Supplementary file 1 [file pharmaceuticals-16-00177-s001.zip › pharmaceuticals-2094684-supplementary.pdf]

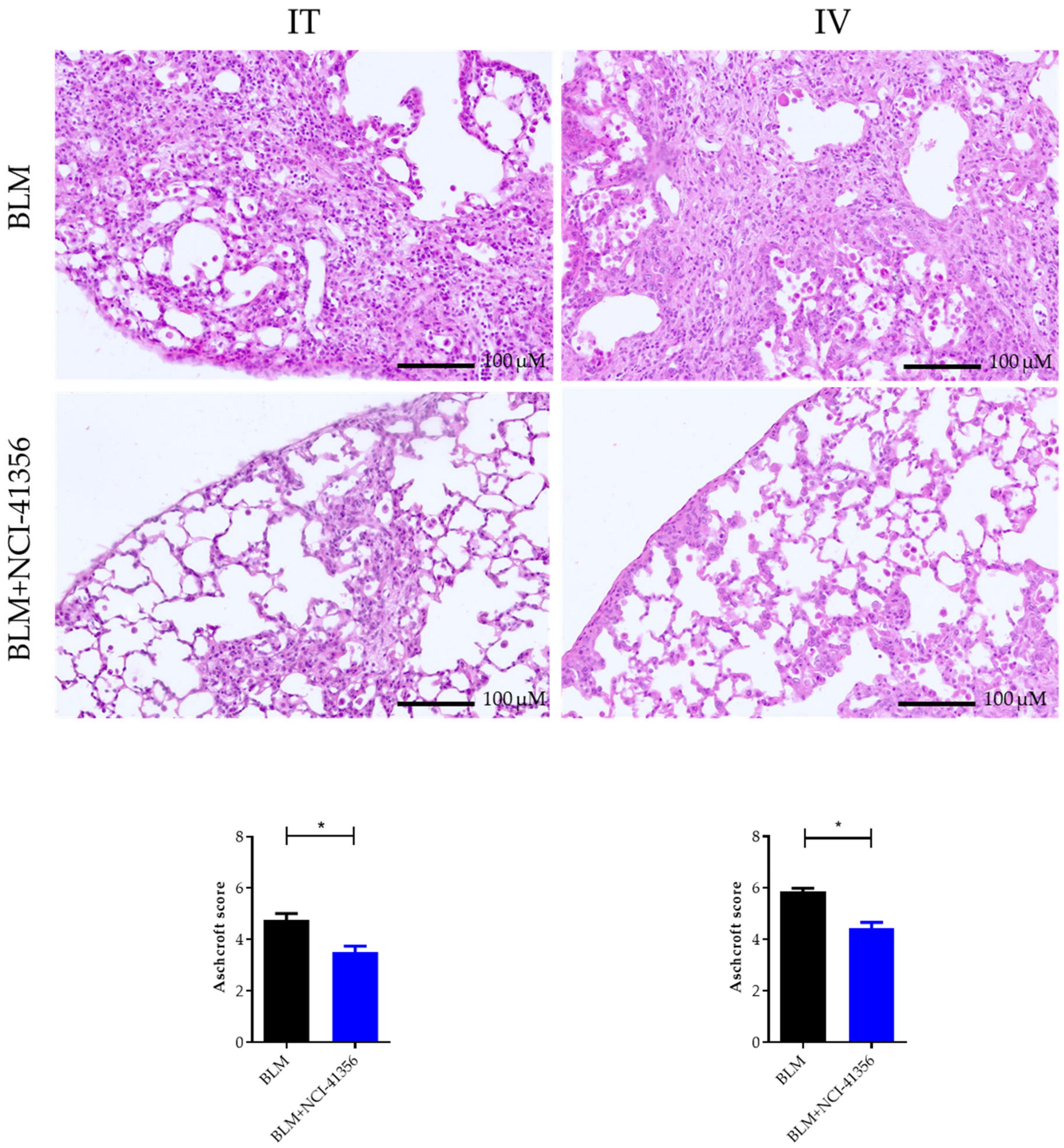

Supplemental Figure S1. Ashcroft analysis of pulmonary fibrosis induced in bleomycin-treated mice treated or not with NCI-41356 by intra-tracheal (IT) or intravenous (IV) routes of administration.

Top Left: Representative images of Haematoxylin and Eosine (H&E) staining of bleomycin-treated mice lungs treated or not with NCI-41356 (15 mg/kg) by intra-tracheal administration (×20) and down left : Ashcroft score of evaluation of fibrosis of the same experimentation (n = 5 /group).

Top right: Representative images of Haematoxylin and Eosine (H&E) staining of bleomycin-treated mice lungs treated or not with NCI-41356 (15 mg/kg) by intravenous administration) (×20) and down right: Ashcroft score of evaluation of fibrosis of the same experimentation (n = 5/group).

Ashcroft scores are expressed as means ± SEM and compared using nonparametric Mann–Whitney test (B). * $p = 0.0159$.

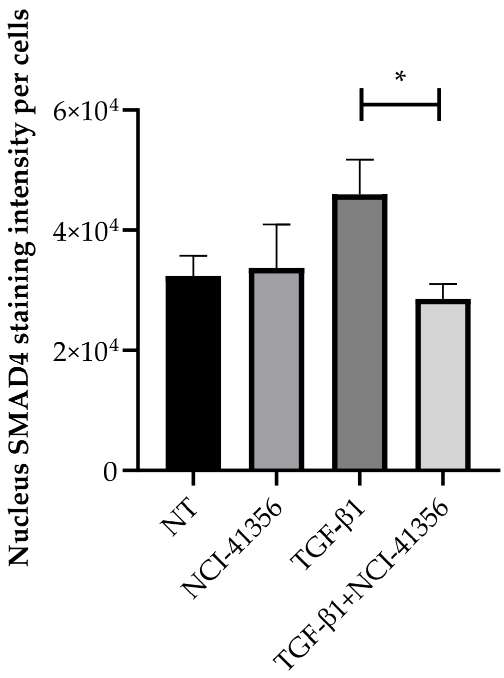

Supplemental Figure S2. Immunofluorescence nucleic SMAD4 quantification evaluated on cells stimulated by TGF-β1 and treated by NCI-41356.

SMAD4 nucleic protein level intensity on cells treated by NCI-41356 (100 μM), TGF-β1 (10 ng/mL) or both compounds. Intensity are expressed as means ± SEM and compared using nonparametric Mann–Whitney test. (* $p = 0.02$).
